# Associations between cerebral magnetic resonance imaging infarct volume and acute ischemic stroke etiology

**DOI:** 10.1371/journal.pone.0256458

**Published:** 2021-08-23

**Authors:** Nicholas Omid Daneshvari, Michelle Christina Johansen

**Affiliations:** 1 Department of Neurology, The Johns Hopkins University School of Medicine, Baltimore, Maryland, United States of America; 2 Department of Neurology, Cerebrovascular Division, The Johns Hopkins University School of Medicine, Baltimore, Maryland, United States of America; Instituto Mexicano del Seguro Social (IMSS) HGZ 2, MEXICO

## Abstract

**Background:**

Identifying ischemic stroke etiology is necessary for proper treatment and secondary prevention. We sought to define associations between infarct volume and stroke subtypes.

**Materials and methods:**

Inclusion criteria necessitated a Johns Hopkins Hospital inpatient admission (2017–2019) for ischemic stroke with confirmatory brain magnetic resonance imaging. Infarct volume was calculated using MRIcron© by a masked reviewer. Ischemic strokes were adjudicated using TOAST classification. Multivariable/multinomial logistic regression determined associations between infarct volume and stroke subtypes with interaction terms for infarct number and location. Stepwise adjustment accounted for potential confounders.

**Results:**

Patients (N = 150) were on average 61 years old, male (58%), and black (57%). Each 5mL increase in infarct volume was associated with cardioembolic (OR 1.07, 95%CI 1.01–1.14) and large-artery occlusions (OR 1.10, 95%CI 1.02–1.18), but lower odds of lacunar stroke (OR 0.18, 95%CI 0.06–0.55). There was no difference in risk of cardioembolic (base) and large-artery atherosclerotic strokes with increasing infarct volume (RRR 1.01, 95%CI 0.94–1.09), but risk of lacunar stroke was decreased (RRR 0.17, 95%CI 0.06–0.53). Infarct number (single vs multiple) modified the association between volume and subtype for large-artery occlusions (p-interaction 0.09).

**Conclusions:**

In this study, larger volume infarcts were significantly associated with both cardioembolic and large-artery atherosclerotic strokes (no difference in the degree of association) and decreased odds of lacunar stroke. A single, large-volume stroke was associated with large-artery atherosclerosis, while multiple infarcts were associated with cardioembolism. Given the differential associations between volume, number of lesions, and stroke etiology, defining stroke subtypes in light of infarct volume might aid in clinical practice.

## Introduction

Ischemic strokes with larger infarct volumes are associated with poor patient outcomes [1–3]. However, the relationship between infarct volume and clinical severity is complex, and this association is likely influenced by stroke etiology [4, 5]. Identification of stroke etiology is necessary for proper treatment [6–8], yet the cause of the stroke is never diagnosed in approximately 30% of patients [9]. Among patients with these strokes of unknown origin, the 10-year rate of recurrence is approximately 32%, potentially attributable to limitations in administering appropriate secondary prevention [10]. Currently, infarct volume is incorporated into the definition of only lacunar stroke and thus not used to distinguish stroke subtypes in the classical definitions. Additionally, rapid cerebral perfusion imaging with consideration of the volume of infarcted brain tissue is increasingly used to make acute treatment decisions for those with large-artery occlusion who may benefit from intra-arterial thrombectomy. As brain imaging in the acute and subacute period is routinely ordered for stroke, and imaging technology is rapidly evolving, infarct volume is an easily estimated parameter that could potentially aid stroke etiology identification. Therefore, appreciating associations between infarct volume and stroke subtype, in addition to the known association with lacunar stroke, would facilitate patient management.

The objective of this study is to define the association between ischemic stroke infarct volume, as well as other infarct imaging characteristics, and stroke subtype in order to determine the utility of these imaging parameters as defining characteristics of non-lacunar strokes. We hypothesize that large, multifocal infarcts are associated with cardioembolic stroke rather than any other stroke subtype. We also explore whether the location or number of lesions could modify the relationship between infarct size and stroke etiology.

## Materials and methods

### Standard protocol approvals, registrations, and patient consents

The Johns Hopkins Hospital (JHH) institutional review board approved the study and all participants provided informed consent for the parent study.

### Study population

The study population for this analysis was part of a larger, ongoing parent study that was conducted at JHH, a comprehensive stroke center and tertiary care, academic hospital. The parent study, the Investigation of Left Atrial Structure and Stroke Etiology (I-LASER), is a prospective cohort study seeking to determine associations between left atrial structure and function, and ischemic stroke subtypes, focusing on cardioembolic and cryptogenic strokes [11]. Inclusion criteria included a JHH inpatient admission between 2017–2019 for acute ischemic stroke, defined clinically by neurological examination and confirmed radiographically on brain magnetic resonance image (MRI) with contrast. Patients were adults (≥18 years old) and had a clinical indication for a transthoracic echocardiogram. Demographic data were collected on admission.

### Stroke infarct volume calculation

Patients underwent a clinically indicated brain 3-T MRI with contrast as part of their stroke care. All sequences included an Axial T1, Axial Diffusion-Weighted Image (DWI), Axial T2* GRE, and Axial T2 FLAIR. Infarct volumes were calculated by manually tracing hyperintense areas of diffusion restriction on patient T2 DWIs using MRIcron© (NeuroImaging Tools & Resources Collaboratory) by a reviewer masked to patient characteristics and stroke subtypes. Such techniques, collectively known as voxel-based morphometry, have been well-validated for calculating infarct volumes in similar research [12, 13]. 30% of the MRI traces were re-reviewed by another reviewer, masked to patient characteristics, to determine interrater reliability, which was greater than 90%.

All scans were normalized to a reference scan to standardize voxel size, number of axial slices, and resolution. Volumes were calculated using Statistical Parametric Mapping 12 (SPM12; Wellcome Center for Human Neuroimaging, London, UK), which operates using MATLAB R2007a (7.4) to R2019b (9.7) (Mathworks, Natick, MA, USA). For analysis, all infarct volumes were converted from mm^3^ to milliliters (mL), and a 5 mL increment was used when reporting results.

### Stroke subtype adjudication

**Fig 1:**
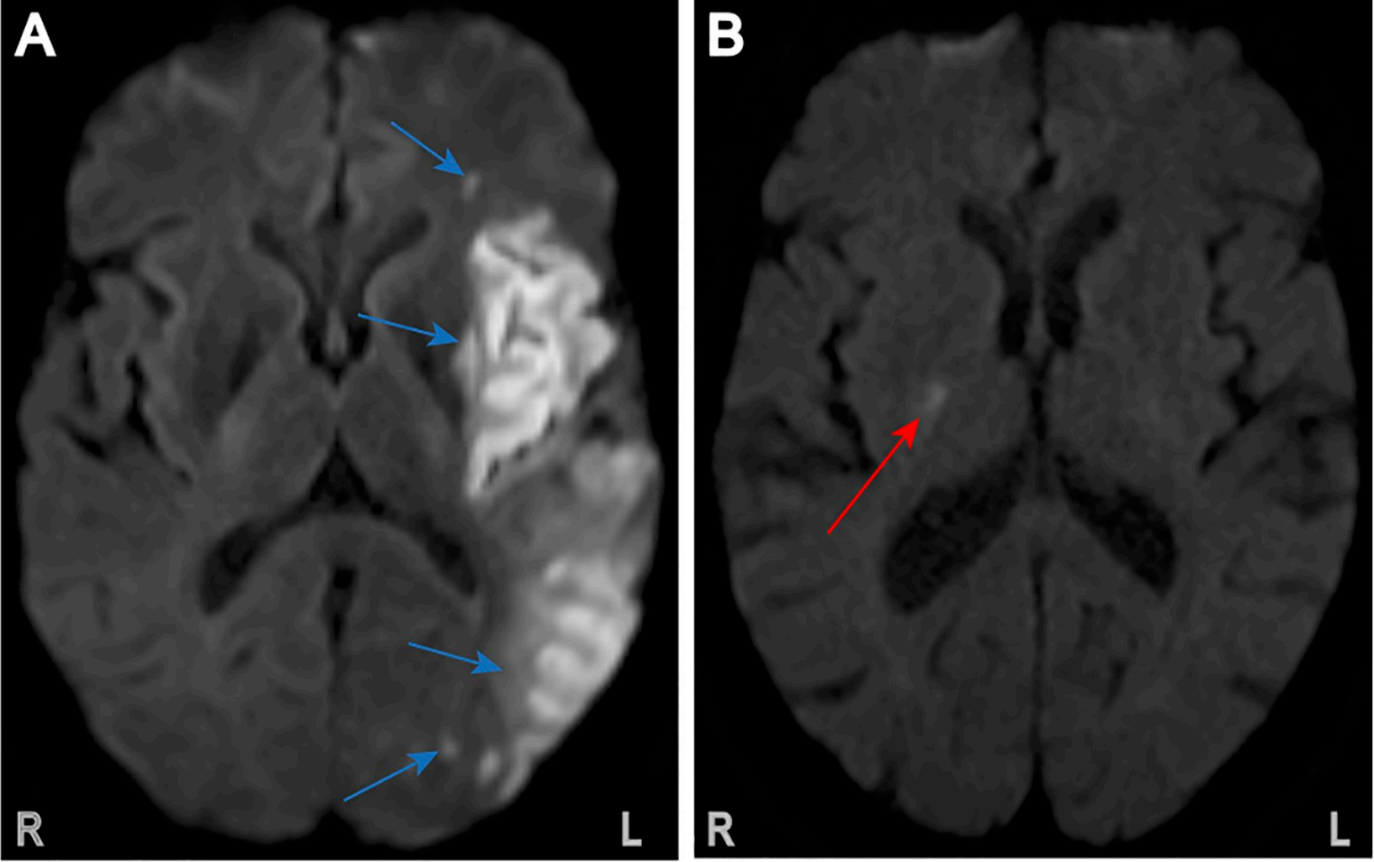
Sample patient brain magnetic resonance images demonstrating acute ischemic stroke patterns on T2 diffusion-weighted images. (A) Adjudicated cardioembolic stroke with multiple lesions (blue arrows) in left middle cerebral artery distribution. Total calculated infarct volume: 105.4mL. (B) Adjudicated small-vessel occlusion stroke with single lesion (red arrow) in the posterior limb of the right internal capsule. Total calculated infarct volume: 0.194mL.

Patients underwent a standard-of-care workup to determine stroke etiology, including brain MRI, cervical vascular imaging, and cardiac monitoring, as determined by the inpatient stroke team, who were not aware of the study. Ischemic stroke subtype was adjudicated according to Trial of Org 10172 in Acute Stroke Treatment (TOAST) criteria by a cerebrovascular neurologist masked to infarct volumes [14]. The TOAST criteria categorize acute ischemic stroke into 5 subtypes: large-artery occlusion, cardioembolic stroke, small-vessel occlusion (lacune), stroke of other determined etiology, and stroke of undetermined etiology [14]. Since the definition of lacune typically includes infarct volume [15], in order to maintain masking, neuroimaging reports were reviewed only to see whether the report mentioned lacunar, small-vessel disease, or other similar description and whether the lesion was in a typical location (basal ganglia, brainstem, thalamus, internal capsule, cerebral white matter) to be considered diagnostic of lacunar infarction. Two representative patient brain MRIs demonstrating cardioembolic and small-vessel strokes are depicted in Fig 1.

Strokes were also characterized by number of infarcts and neuroanatomical location. Number of infarcts was categorized as single versus multiple. A single lesion was defined radiographically as a contiguous hyperintensity in one vascular territory, whereas multiple infarcts were two or more, distinguishable lesions separated in space. Infarct location was defined as infratentorial, supratentorial, or both.

### Statistical analysis

Prospectively collected data from the parent study were analyzed for this cross-sectional analysis as follows. The primary dependent variable was stroke subtype, and the primary independent variable was infarct volume. Infarct volume was scaled so that the effect estimates are per 5 mL increment. Infarct volume was not standardized to enable volumes to be compared across stroke subtype. Covariates of interest were age, sex, race, body mass index (BMI; kg/m^2^), low-density lipoprotein (LDL; mg/dL), hemoglobin A1C (HgA1C; %), systolic blood pressure measured on admission (SBP; mmHg), and smoking history. Race was categorized as black vs other. Smoking history was defined as ever-smoker versus never-smoker. Multivariable logistic regression was used to determine the association between infarct volume and each stroke subtype. Multinomial logistic regression further compared the relative risk of cardioembolic stroke (base outcome) versus large-artery atherosclerosis or small-vessel disease stroke. Stepwise adjustment accounted for potential confounders as follows: Model 1 –age, sex, and race; Model 2 –Model 1 + SBP, BMI, LDL, HgA1C, and smoking history. Potential confounders were chosen a priori based on published literature [16–21].

Interaction terms were included where appropriate to determine whether associations between infarct volume and stroke subtype was modified by infarct number or location. Finally, a sensitivity analysis was performed by excluding the two largest stroke infarcts, both due to large-artery atherosclerosis, that were determined to be statistical outliers. All analyses were performed using Stata v14.1 and Stata v15.1 [22, 23]. Effect estimates were defined using 95% confidence intervals (CIs) with statistical significance defined as p < 0.05 or an interaction p value < 0.1.

## Results

### Patient characteristics

**Fig 2:**
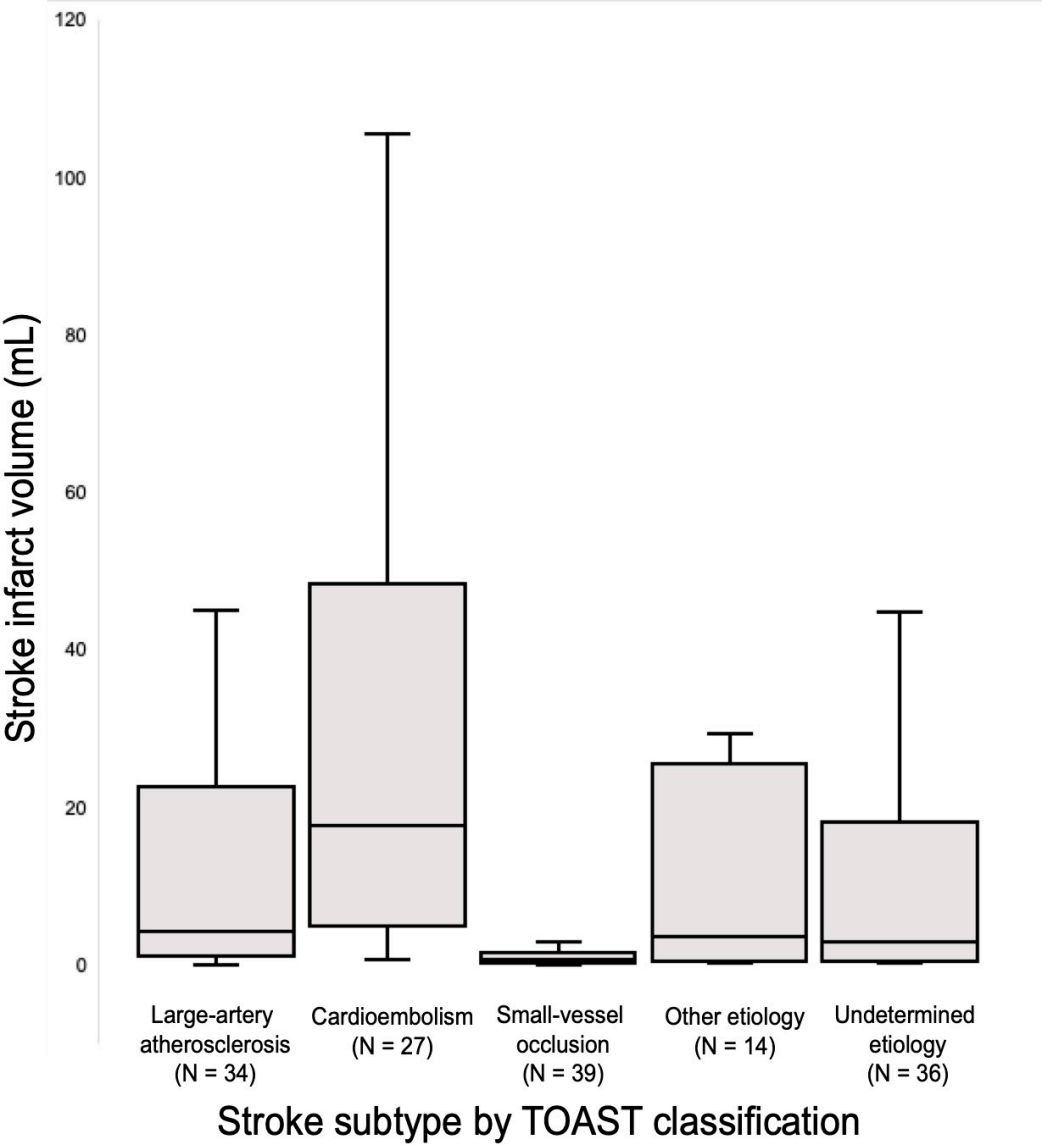
Box and whisker plots of stroke infarct volume across different stroke etiologies.

150 patients met inclusion criteria, of whom 27 were adjudicated as having cardioembolic strokes (18%), 34 large-artery occlusions (22.7%), 39 small-vessel occlusions (26%), 14 strokes of other etiology (9.33%), and 36 strokes of unknown causes (24%). Mean infarct volumes (± standard deviation) by stroke subtype were as follows: 28.88 ± 28.98 mL cardioembolic, 24.31 ± 47.53 mL large-artery atherosclerosis, 1.34 ± 1.89 mL small-vessel occlusion, 11.88 ± 18.49 mL other etiology, 11.77 ± 17.54 mL strokes of unknown origin (Fig 2). Of the 150 patients, 65 (43%) had strokes confined to a single lesion, while 85 (57%) had multiple infarcts. Analysis of infarct location revealed that 27 (18%) were infratentorial, 109 (73%) were supratentorial, and 14 (9%) were both above and below the tentorium. Cardioembolic strokes and large-artery atherosclerotic strokes tended to be located supratentorially (cardioembolic 23/27 [85%]; large-artery occlusion 26/34 [76%]), while small-vessel strokes were more widely distributed (infratentorial 13/39 [33%]; supratentorial 24/39 [62%]; both 2/39 [5%]).

On average, the patients were 61 years old (range 18–98 years), and the majority were male (N = 87, 58%) and black (N = 86, 57.3%; Table 1). The average patient in this population was pre-diabetic (mean HgA1C = 6.37 ± 1.97%), overweight (mean BMI = 29 ± 7 kg/m^2^), and hypertensive (mean SBP = 151.73 ± 32.3 mmHg). Nearly half of patients (N = 67, 44%) either currently smoked, or had smoked during their lifetime. Compared to patients with all other stroke subtypes, those with cardioembolic stroke were more likely to have higher BMIs (cardioembolic 31.9 ± 8.1 kg/m^2^, all others 28.5 ± 6.6 kg/m^2^, p = 0.02). The mean LDL for cardioembolic stroke patients was lower (cardioembolic 91.0 ± 36.0 mg/dL, all others 107.7 ± 43.2 mg/dL), but this finding was not statistically significant (p = 0.06).

**Table 1:** Demographic characteristics and ischemic stroke risk factors of patients at baseline (N = 150).

**Age, years**	61 (14)*
**Male sex, n (%)**	87 (58)
**Race,** ^**a**^ **n (%)**	86 (57)
**Body mass index, kg/m** ^**2**^	29.1 (7.0)
**Low-density lipoprotein, mg/dL**	104.65 (42.37)
**Hemoglobin A1C, %**	6.37 (1.96)
**Systolic blood pressure, mmHg**	151.73 (32.23)
**Smoking history,** ^**b**^ **n (%)**	67 (44)

Mean (SD) unless otherwise specified.

^a^Race: black vs other

^b^Smoking history: ever smoker vs never smoker

### Associations between infarct volume and stroke subtype

Per 5 mL increase in infarct volume, there was a significantly higher odds of having a cardioembolic stroke (OR 1.07, 95% CI 1.01–1.14, model 2) versus all other subtypes after adjusting for demographics and vascular risk factors (Table 2). There was approximately a 10% increased odds of large-artery occlusion per 5 mL increase in infarct volume (OR 1.10, 95% CI 1.02–1.18, model 2). By contrast, the odds of small-vessel stroke was over 5 times lower with the same incremental increase in infarct volume (OR 0.18, 95% CI 0.06–0.55, model 2).

**Table 2:** Multivariable logistic regression demonstrating the adjusted odds ratio (OR) of each specified stroke subtype per 5 mL increase in ischemic stroke infarct volume (N = 150).

Stroke Subtype	Model 1			Model 2		
	OR	95% CI	p-value	OR	95% CI	p-value
**Cardioembolic**	1.08	1.02–1.15	0.02	1.07	1.01–1.14	0.03
**Large-artery atherosclerosis**	1.07	1.00–1.14	0.04	1.10	1.02–1.18	0.01
**Small-vessel occlusion**	0.20	0.07–0.57	0.002	0.18	0.06–0.55	0.003

^a^Abbreviations: OR = odds ratio, CI = confidence interval.

^b^Adjustment model 1: Age, sex, and race (black vs other); Model 2: Model 1 + systolic blood pressure (mmHg), body mass index (kg/m^2^), hemoglobin A1C (%), low-density lipoprotein (mg/dL), and smoking history (ever smoker vs never smoker).

^c^Mean infarct volumes (± standard deviation): cardioembolic 28.88 ± 28.98 mL, large-artery atherosclerosis 24.31 ± 47.53 mL, small-vessel occlusion 1.34 ± 1.89 mL, other etiology 11.88 ± 18.49 mL, unknown origin 11.77 ± 17.54 mL.

When compared directly, there was no significant difference in the relative risk of a cardioembolic versus a large-artery atherosclerotic stroke per unit increase in infarct volume (RRR 1.01, 95% CI 0.94–1.09, base cardioembolic) (Table 3). However, the relative risk of small-vessel occlusion was 83% lower (RRR 0.17, 95% CI 0.06–0.53, base cardioembolic).

**Table 3:** Multinomial logistic regression demonstrating the adjusted relative risk ratio (RRR) of each specified stroke subtype (reference: cardioembolic) per 5 mL increase in ischemic stroke infarct volume (N = 150).

Stroke Subtype	RRR	95% CI	p-value
**Cardioembolic (reference)**	-	-	-
**Large-artery atherosclerosis**	1.01	0.94–1.09	0.72
**Small-vessel occlusion**	0.17	0.06–0.53	0.002

^a^Abbreviations: RRR = relative risk ratio, CI = confidence interval.

^b^Adjustment model 2: Age, sex, race (black vs other), systolic blood pressure (mmHg), body mass index (kg/m^2^), hemoglobin A1C (%), low-density lipoprotein (mg/dL), and smoking history (ever smoker vs never smoker).

^c^Mean infarct volumes (± standard deviation): cardioembolic 28.88 ± 28.98 mL, large-artery atherosclerosis 24.31 ± 47.53 mL, small-vessel occlusion 1.34 ± 1.89 mL, other etiology 11.88 ± 18.49 mL, unknown origin 11.77 ± 17.54 mL.

### Effect measure modification of stroke subtype with number and location of infarcts

There was a significant interaction between infarct volume and number of infarcts among patients with strokes due to large-artery atherosclerosis (p-interaction 0.09, Table 4). Specifically, per 5 mL increase in infarct volume, strokes confined to a single lesion had a higher odds of being adjudicated as large-artery occlusion (single OR 1.11, 95% CI 1.01–1.23). There was a suggestion of there being an interaction between infarct volume and number of infarcts for cardioembolic strokes (p-interaction 0.13), with cardioembolism being significantly associated with multiple infarcts, but we interpret this cautiously. There was no evidence of a difference in the association between infarct volume and number of infarcts among those with small-vessel occlusion. In terms of effect measure modification by stroke location, sample size was insufficient for formal interaction terms, as the majority of all strokes were supratentorial.

**Table 4:** Effect measure modification by number of infarcts demonstrating the adjusted odds ratio (OR) of each specified stroke subtype per 5 mL increase in ischemic stroke infarct volume (N = 150).

Stroke Subtype	Single Infarct			Multiple Infarcts			Interaction		
	OR	95% CI	p-value	OR	95% CI	p-value	OR	95% CI	p-value
**Cardioembolic**	1.04	0.96–1.12	0.32	1.15	1.03–1.28	0.01	1.10	0.96–1.12	0.13
**Large-artery atherosclerosis**	1.11	1.01–1.23	0.03	0.98	0.89–1.09	0.77	0.88	0.77–1.02	0.09
**Small-vessel occlusion**	0.32	0.10–1.03	0.06	0.42	0.13–1.38	0.15	1.30	0.25–6.85	0.76

^a^Abbreviations: OR = odds ratio, CI = confidence interval.

^b^Adjustment model 2: Age, sex, race (black vs other), systolic blood pressure (mmHg), body mass index (kg/m^2^), hemoglobin A1C (%), low-density lipoprotein (mg/dL), and smoking history (ever smoker vs never smoker).

^c^Mean infarct volumes (± standard deviation): cardioembolic 28.88 ± 28.98 mL, large-artery atherosclerosis 24.31 ± 47.53 mL, small-vessel occlusion 1.34 ± 1.89 mL, other etiology 11.88 ± 18.49 mL, unknown origin 11.77 ± 17.54 mL.

### Sensitivity analysis excluding two strokes with largest infarct volumes

The two largest infarcts, statistical outliers both due to large-artery atherosclerosis, were excluded from the dataset and the primary analysis was repeated. The odds of cardioembolic stroke per unit increase in infarct volume remained elevated with an increased effect estimate (OR 1.18, 95% CI 1.07–1.30, model 2). There was a significantly lower odds of having a small-vessel disease stroke as before (OR 0.18, 95% CI 0.06–0.55). Large-artery atherosclerotic strokes maintained a similar effect estimate, but the association was no longer statistically significant (OR 1.05, 0.95–1.17, model 2).

## Discussion

In this single-center prospective cohort study, larger volume infarcts were associated with cardioembolic or large-artery atherosclerotic strokes, after adjusting for patient demographics and vascular risk factors. When comparing the risk of cardioembolic to large-artery atherosclerotic stroke by volume, this study did not detect a difference. However, larger volume strokes confined to a single lesion were associated with large-artery occlusion, while multiple infarcts exhibited a trend-level association with cardioembolic stroke. In keeping with the classic definition of a lacune, this study confirmed a decreased odds of small-vessel occlusion with higher infarct volumes [15].

Small-vessel disease strokes typically involve smaller brain structures, and therefore have lower volumes as considered in the TOAST definition; our findings are supportive of this conclusion. However, unlike with lacunar strokes, infarct volume is not incorporated into the definitions of either cardioembolic or large-artery occlusions. Although particular patterns of imaging findings are considered suggestive of one stroke etiology (for example, proximal embolic versus single-artery occlusion), these patterns are not conclusive and only aid in diagnosis. Because infarct volume is easily estimable on routine imaging studies, modifying stroke subtype definitions with regards to volume could help guide decision making in the setting of acute ischemic stroke.

Currently, stroke providers incorporate imaging findings to rapidly assess the volume of infarcted tissue. Providers also use imaging metrics to aid decision making, such as the Alberta Stroke Program Early CT Score (ASPECTS) when deciding whether to pursue acute endovascular thrombectomy in the setting of large-vessel occlusion [24]. However, stroke etiology is not directly considered in this decision. Given that neuroimaging has and continues to rapidly evolve (e.g., perfusion imaging), utilizing these images to also facilitate diagnosis of stroke etiology could assist decisions regarding secondary prevention once the acute treatment window is over.

In our work, we found that larger volume infarcts confined to a single lesion were associated with increased odds of large-artery occlusion, whereas high-volume strokes with multiple infarction sites tended to be cardioembolic in origin. Since cardioembolic and large-artery atherosclerotic strokes were indistinguishable by infarct volume in this study, additional imaging characteristics such as number of lesions may be more useful to incorporate into future radiographic determination of stroke etiology. Given that these two stroke subtypes usually require different management to prevent recurrent stroke [7], accurate diagnosis is imperative, suggesting value in supplementing stroke subtype definitions with these imaging parameters.

On our repeat analysis excluding the two largest infarcts, which were outliers and both due to large-artery occlusion, there was no longer a significant association between infarct volume and large-artery occlusion. One possible explanation is that large-artery atherosclerosis infarct volumes display a multimodal distribution with a small peak at very high volumes. Further research is needed to determine whether these outliers were due to chance or represent a meaningful pattern.

Stroke etiology remains unknown for approximately 30% of patients [9], about one in three of whom suffers a recurrent stroke within 10 years [10]. Infarct volume and radiographic imaging characteristics may be one additional tool by which physicians can identify the cause of the stroke. The association between patient outcomes and stroke volume is nonlinear. While larger volume infarcts are typically associated with worse outcomes [1, 2], patients with lacunar stroke, by definition, have small infarcts, yet 20–30% can become severely disabled, particularly with regards to motor function [5, 25]. A further understanding of the relationships between volume and outcomes could likely be obtained with increased consideration of stroke etiology, improving clinician ability to predict patient prognoses.

We recognize that there are limitations to this study. The inclusion criteria requiring a clinical indication for transthoracic echocardiogram, though standard practice for most comprehensive stroke centers, may limit generalizability to all populations. The sample size may have been insufficient to detect meaningful differences in subgroup analyses. Finally, we chose to exclude two outliers in a sensitivity analysis; though these outliers may have created a type 1 error, it is also possible that these large strokes represent a natural phenomenon, hence their inclusion in the primary analyses.

## Conclusions

We have shown that larger volume infarcts were more likely to be cardioembolic or large-artery atherosclerotic strokes, but less likely due to small-vessel occlusion. Higher-volume strokes confined to a single lesion were more often secondary to large-artery occlusions, whereas those with multiple lesions tended to be cardioembolic in origin. Findings such as these suggest possible value in viewing infarct volume and number of lesions as meaningful components of stroke subtypes. Given that identification of ischemic stroke subtype is necessary for optimal treatment and secondary prevention [6–8], we suggest that these imaging parameters could be operationalized to help identify stroke etiology.

## Supporting information

S1 Dataset (XLSX)
